# Ontogeny and phylogeny: molecular signatures of selection, constraint, and temporal pleiotropy in the development of *Drosophila*

**DOI:** 10.1186/1741-7007-7-42

**Published:** 2009-07-21

**Authors:** Carlo G Artieri, Wilfried Haerty, Rama S Singh

**Affiliations:** 1 Department of Biology, McMaster University, Hamilton, Ontario, Canada

## Abstract

**Background:**

Karl Ernst Von Baer noted that species tend to show greater morphological divergence in later stages of development when compared to earlier stages. Darwin originally interpreted these observations via a selectionist framework, suggesting that divergence should be greatest during ontogenic stages in which organisms experienced varying 'conditions of existence' and opportunity for differential selection. Modern hypotheses have focused on the notion that genes and structures involved in early development will be under stronger purifying selection due to the deleterious pleiotropic effects of mutations propagating over the course of ontogeny, also known as the developmental constraint hypothesis.

**Results:**

Using developmental stage-specific expressed sequence tag (EST) libraries, we tested the 2 hypotheses by comparing the rates of evolution of 7,180 genes obtained from 6 species of the *Drosophila melanogaster *group with respect to ontogeny, and sex and reproduction-related functions in gonadal tissues. Supporting morphological observations, we found evidence of a pattern of increasing mean evolutionary rate in genes that are expressed in subsequent stages of development. Furthermore, supporting expectations that early expressed genes are constrained in divergence, we found that embryo stage genes are involved in a higher mean number of interactions as compared to later stages. We noted that the accelerated divergence of genes in the adult stage is explained by those expressed specifically in the male gonads, whose divergence is driven by positive selection. In addition, accelerated gonadal gene divergence occurs only in the adult stage, suggesting that the effects of selection are observed primarily at the stages during which they are expected occur. Finally, we also found a significant correlation between temporal specificity of gene expression and evolutionary rate, supporting expectations that genes with ubiquitous expression are under stronger constraint.

**Conclusion:**

Taken together, these results support both the developmental constraint hypothesis limiting the divergence of early expressed developmentally important genes, leading to a gradient of divergence rates over ontogeny (embryonic < larval/pupal < adult), as well as Darwin's 'selection opportunity' hypothesis leading to increased divergence in adults, particularly in the case of reproductive tissues. We suggest that a constraint early/opportunity late model best explains divergence over ontogeny.

## Background

For over a century, developmental biologists have noted an ontogenic pattern among evolutionary relationships: earlier developmental stages are morphologically more similar across species than later stages; this is also known as Von Baer's third law [1-4]. While more recent studies in vertebrates have determined that the very earliest stages of ontogeny (for example, gastrulation) may be subject to substantial variation even among closely related species, upon reaching the tailbud stage, embryos begin to share more similarity in appearance that gradually declines with subsequent development [5]. This 'hourglass' model of developmental similarity among vertebrates suggests that, while certain stages of development undergo substantial change over evolutionary time, there exists a significant conservation of the mechanisms underlying development across vertebrates [6-9]. Darwin originally interpreted Von Baer's observations via a selectionist framework [10,11]. He suggested that divergence should be greatest during ontogenic stages in which organisms experienced the most varying 'conditions of existence' and, as a result, occasioned opportunity for differential selection [2]. Embryos of varied species are therefore more similar than adults due to exposure to very similar fetal environments. Furthermore, he noted that derived features rarely appeared in an organism before the stage when they were used, indicating that the effect of selection was also specific to the stage where selection pressure actually occurred. This observation was important to his overall hypothesis, as selection pressures occurring during one stage that selected for traits expressed in other stages would be inconsistent with Von Baer's observations. Using secondary sexual traits as a primary example, Darwin compiled a large number of observations indicating that male-specific structures known to be highly divergent even among closely related species rarely developed until reproductive maturity was reached [10,12].

Modern interpretations of Von Baer's third law have focused on another, non-mutually exclusive mechanism: genes implicated in early aspects of development are more likely to regulate a large number of downstream effectors via hierarchical regulatory cascades, and are thus more evolutionarily constrained due to the large deleterious pleiotropic effects of mutations. This is known as the developmental constraint hypothesis [3,13,14]. The complex hierarchical nature of gene regulatory networks has become a focus of major interest in the field of organismal development [15,16] with special attention being paid in particular to those network modules critical to early development and conserved over broad evolutionary distances [17]. For instance, the well known homeotic genes involved in establishing the anterior/posterior axis in the early development of most metazoans provide a striking example of highly conserved genes whose mutations are known to have extensive pleiotropic consequences [18-20]. These transcription factors are also known to act as master regulatory switches in cascades involved in regulating the proper expression of many downstream, developmentally important effectors [21]. Another example is the gene regulatory feedback loop required for endoderm specification in echinoderms, which encodes several transcription factors whose inactivation has catastrophic effects on the entire body plan [17,22]. These instances highlight the strength of purifying selection acting on specific genes known to be involved in complex developmental regulatory networks; however a more recent interest has concerned the broader evolutionary patterns of the genome with respect to ontogeny.

The evolutionary dynamics of genes expressed over the course of development have recently been examined at the genomic level in the case of flies and nematodes, using microarray-based information about the developmental timing of gene expression [23-25]. Castillo-Davis and Hartl [23] used previously published, developmental stage-specific microarray data [26] in order to compare the rates of coding sequence divergence of a relatively small number of genes (224) between *Caenorhabditis elegans *and *C. briggsae *(20 million to 120 million years diverged (MYD)). Genes in their dataset were classified either as 'non-modulated' genes (that is, invariant in expression level over development), early-expressed genes (that is, embryonic), or late-expressed genes (that is, larval and adult) based on the developmental stage at which their peak level of expression occurred. The authors found no significant difference in the rates of protein evolution among the three categories, though the early-expressed genes showed a higher rate of synonymous substitution as well as a lower codon usage bias (CUB) than late-expressed genes. The analysis of the same 2 species was subsequently refined by Cutter and Ward [24] using a larger dataset of 7,281 genes and a larger source of developmental expression data [27,28]. Their results support some theoretical predictions of both the developmental constraint as well as Darwin's 'selection opportunity' hypothesis: when genes were classified based on the stage at which their peak expression level occurred, adult genes were found to be evolving more rapidly than those in the earlier, larval stage. Expression level in the larval stage, relative to the adult, was also found to be negatively correlated with sequence divergence, while the opposite was observed for expression in adults. However, the authors noted no unidirectional trend in evolutionary rates in genes expressed over the course of embryogenesis, as would be predicted by the developmental constraint hypothesis, leading them to suggest that constraint may not explain the evolutionary rates of proteins expressed during embryonic development in these species. Furthermore, when examining the tissue specificity of genes expressed in adult nematodes, the authors found that the majority, though not all, of the acceleration in evolutionary rate observed in this stage was explained by genes expressed primarily in the male gametes, providing evidence of a significant effect of sexual selection, presumably acting through sperm competition between males and hermaphrodites or antagonistic coevolution between genes expressed in sperm and oocytes [24].

Davis *et al*. [25] used the results of a microarray study of the expression levels of 4,028 genes over the course of *Drosophila melanogaster *ontogeny [29] and examined their rates of sequence divergence between *D. melanogaster *and *D. pseudoobscura *(25 to 55 MYD). They noted that gene expression level in the late embryo relative to later stages was negatively correlated with sequence divergence, while the opposite was observed in the case of adult males. However, the authors noted no significant correlation between expression levels and sequence divergence for the many of the sampled developmental stages. Unfortunately the species pairs used in both of these studies were quite distantly diverged and thus interpretation of these data is limited due to the saturation of synonymous site divergence (*d*_S_), which largely prevents investigation of questions regarding evidence of selection [30,31]. Furthermore, comparisons at such evolutionary distances allow the possibility that expression patterns (for example, time of expression, sex bias, and so on) have diverged between species, questioning whether similar selective pressures are acting along both lineages at the level of individual genes [32].

Holometabolic insects such as *Drosophila *provide an excellent model for studying gene evolution over ontogeny as they pass through four separate, unambiguous developmental stages (embryo, larva, pupa, and adult). A large body of information about the evolutionary dynamics of the genomes of drosophilids has accumulated, aided significantly by the recent release and analysis of the complete genomes of 12 *Drosophila *species [33]. However, the relationship between development and genomic evolution remains largely unexplored. Here, we analyze a larger dataset than was previously available, using information generated from publicly available developmental stage-specific expressed sequence tag (EST) libraries to assign genes to specific developmental stages and determine their evolutionary patterns within the *D. melanogaster *group, allowing more reliable estimates of divergence parameters as well as reducing the caveats associated with comparing distantly related species [25]. We report a gradient of increasing mean evolutionary rate in genes expressed in subsequent stages of fly development, culminating in exaggerated gene sequence divergence specifically in adult males. When comparing genes expressed specifically in the gonads of embryos to adults, we found that the increased rate of divergence observed in adults is explained entirely by those genes expressed in the testis. No such pattern of accelerated gene divergence is observed in the embryonic gonads, supporting Darwin's expectations that selection pressures should act predominantly in the stage where the opportunity for selection occurs [10]. Finally, when classifying genes into specific developmental stages using a series of increasing stage-specificity thresholds, we found a significant correlation between specificity of temporal stage of expression and evolutionary rate. We also reanalyzed the dataset used by Davis *et al*. [25] using our methods in order to refine their estimates of divergence and test the generality of their results (Additional files 1, 2, and 3). Taken together, our results support both developmental constraint acting to limit the divergence of early expressed, developmentally important genes [5,8], as well as the notion that accelerated divergence in adults is primarily due to increased selection pressures occurring during this stage.

## Results

### Analysis of the EST library-based developmental profile

We obtained developmental stage specific information for 7,180 genes found in the 6 species of the *D. melanogaster *group (~17 MYD) [34] in the *Drosophila *12 Genomes Consortium dataset [33] from UniGene [35] (see Methods), representing an approximate 2.5-fold increase in size over the *Drosophila *developmental timecourse dataset used by Davis *et al*. [25]. We were unable to obtain separate libraries representing the larval and pupal stages, therefore we pooled all available EST libraries into three developmental stages based on the stage during which they were generated: embryonic, larval/pupal, and adult. Genes were classified into developmental stages based on the stage during which they show their highest proportion of representation in the EST libraries, under the assumption that this represents a biologically reasonable proxy of when the majority of a gene's function(s) occur. However, given that such a method of classification may be subject to complications arising from normal within individual variation in gene expression levels, we reclassified genes using increasing stage-specificity thresholds (see Methods, Table 1 and Additional file 4).

**Table 1:** Number of genes classified into each category according to the proportion of representation specificity thresholds used to classify the expressed sequence tag (EST) data

	Specificity threshold				
	
	None	>Twofold	>Fourfold	>Eightfold	Unique
Stage:					
Embryonic	3,256	2,171	1,342	959	725
Larval/pupal	1,358	739	392	205	100
Adult	2,566	1,834	1,427	1,259	1,191
Stage/tissue – gonads combined:					
Embryonic general	2,234	1,375	855	654	570
Embryonic gonads	905	520	293	117	39
Adult general	1,284	794	593	500	468
Adult gonads	1,688	1,154	817	623	402
Stage/tissue – gonads separated:					
Embryonic general	1,904	1,144	756	636	570
Embryonic gonads	775	415	221	103	39
Adult general	1,138	754	576	496	468
Adult ovary	1,057	503	202	92	27
Adult testis	1,388	1,011	779	617	367

Larval/pupal represents the pooled larval and pupal stages. None: No threshold; >twofold, >fourfold, >eightfold: greater than twofold, fourfold, or eightfold proportion of representation relative to other stages, respectively; Unique: genes that are unique to a single developmental stage (see Methods).

As a test of our assumption that a gene's highest stage of expression is also the stage during which the majority of its functions occur, we performed pairwise comparisons of the lists of genes classified at each stage for each specificity threshold using FatiGO [36,37] (Additional file 5). We found that certain 'biological process' gene ontology (GO) terms associated with temporal-specific functions were consistently over-represented among genes classified into the stage(s) during which such functions were expected to occur. For instance, in the embryogenic versus adult comparison, terms associated with development and regulation (for example, 'regulation of biological process' (GO:0050789), and 'multicellular organismal development' (GO:0007275)) were consistently over-represented among genes classified as embryonic, while terms associated with detection and response to external stimuli were over-represented among genes classified as adult (for example, 'detection of stimulus' (GO:0051606), and 'response to abiotic stimulus' (GO:0009628)). Similar trends were observed in the comparison between the combined larval and pupal stages versus the adult stage, where for example, the term 'post-embryonic development' (GO:0009791) was over-represented among larval/pupal genes, as expected. In the comparison between the embryonic versus larval/pupal stages terms associated with regulation (for example, 'regulation of biological process' (GO:0050789)) tend to be over-represented among embryonic genes while those associated with energy metabolism (for example, 'generation of precursor metabolites and energy' (GO:0006091) and 'carbohydrate metabolic process' (GO:0005975)) tend to be over-represented in the larval/pupal stage, as may be expected given the large amount of organismal growth occurring during the larval stage [38]. Curiously, the term 'sexual reproduction' (GO:0019953) is consistently over-represented among genes classified as being specific to the embryonic and larval/pupal stages as compared to the adult stage (Additional file 5). These genes may be associated with organogenesis of sexual organs, which occurs prior to adulthood, or with spermatogenesis, which begins in the third instar larval stage [39]. However, in general, terms were over-represented in pairwise comparisons in the expected direction, providing support to our assumption of an association between expression level and temporal function.

**Figure 1:**
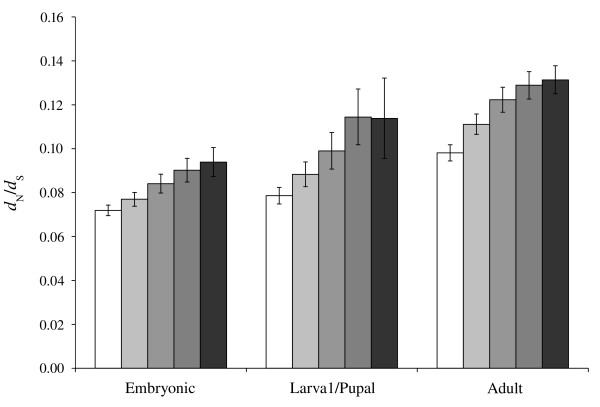
**Average non-synonymous site divergence/synonymous site divergence (*d*_N_/*d*_S_) values for genes classified into developmental stages based on expressed sequence tag (EST) data**. Averages are shown with permuted 95% confidence intervals (95% CIs) for each specificity threshold (from left to right, in increasing contrast): no specificity threshold, greater than twofold, fourfold, or eightfold proportion of representation relative to other stages, and unique to a single developmental stage. Larval/pupal represents the pooled larval and pupal stages. The differences in the distributions between stages within a specificity threshold were found to be statistically significant for most thresholds (*P *< 0.01). Furthermore the differences between thresholds within a stage were also found to be statistically significant in most pairwise comparisons (*P *< 0.05) (Additional file 7).

We found that adult stage genes are evolving more rapidly than earlier stages in non-synonymous site divergence (*d*_N_), *d*_S_, and *d*_N_/*d*_S _at most specificity thresholds (Kruskal-Wallis rank sum test *P *< 0.01; Bonferroni correction was applied to all pairwise comparisons) (Figure 1, Additional file 5). Moreover, at all specificity thresholds, genes classified into the larval/pupal stage experience a higher *d*_N_/*d*_S _than those of the embryonic stage (*P *< 0.05). To our knowledge, this represents the first empirical evidence of a gradient in evolutionary rates spanning the whole of *Drosophila *ontogeny, wherein genes represented at their highest level in the adult are evolving more rapidly than those in the pooled larval and pupal stages, and both are evolving more rapidly than those in the embryonic stage (that is, embryonic < larval/pupal < adult). Under most specificity thresholds, the *d*_N _of larval/pupal genes was also significantly greater than those of embryo genes; however, the *d*_S _of larval/pupal genes was significantly lower than that of embryonic genes, such that the relationship among stages in terms of the *d*_S _was larval/pupal < embryonic < adult (Additional file 6). Previous studies have demonstrated a positive relationship between tissue specificity of expression and evolutionary rate, presumably due to selection against the deleterious pleiotropic effect of mutation restricting the divergence of broadly expressed genes [40-42]. We sought to test for a similar relationship between temporal specificity of gene expression (that is, stage specificity) and the rate of evolution by comparing the mean *d*_N_/*d*_S _between our specificity thresholds at each of our three developmental stage classifications (Figure 1). We performed Bonferroni corrected, pairwise permuted Kruskal-Wallis tests between the distributions of divergence parameters at each of the specificity thresholds within each stage (Additional file 7), and found that there is a clear relationship between the stage specificity of representation in EST libraries and the mean evolutionary rate of genes at that stage: for most comparisons, the more specific a gene's representation at a particular stage, the higher its rate of divergence (*P *< 0.05) (Figure 1). The large confidence intervals associated with the larval/pupal stage are likely due to a reduced number of genes classified as specific to this stage, especially in the case of the higher specificity thresholds (Table 1). Similar results are seen in the case of *d*_N_, however, in the case of *d*_S _there was no significant difference between specificity thresholds (Additional file 8), with the exception of the adult stage, where the highest specificity thresholds have a significantly higher *d*_S _than low specificity thresholds (for example, genes showing greater adult stage specificity tend to have a higher *d*_S_) (Additional file 7).

The selection opportunity hypothesis [10] predicts not only that the average rate of change must increase over developmental stages, but also that the proportion of genes showing evidence of positive selection should increase with subsequent developmental stages [43]. We tested this prediction by performing pairwise comparisons of the proportion of genes showing significant evidence of positive selection using the comparison between models 7 and 8 in phylogenetic analysis by maximum likelihood (PAML) [44] according to the *Drosophila *12 Genomes Consortium data [33], at each stage and for each specificity threshold (Additional file 4). After applying a Bonferroni correction for multiple tests, we found no significant differences in the proportion of genes showing evidence of positive selection in any pairwise comparisons between stages (Additional file 9).

### Stage specificity of selection pressure

A key postulate of the selection opportunity hypothesis [10] is that the effects of late-stage acting selective pressures primarily affect features specific to the stage at which they occur. As a test of this hypothesis, we sought to compare the effect of expression of genes within gonads relative to those expressed in the rest of the body at the two stages in which we had tissue-specific EST library representation information: embryo and adult. Genes were separately classified into either four different stage/tissue categories (embryonic general, embryonic gonads, adult general, and adult gonads) or five tissue categories (wherein the adult gonad library is separated into adult ovary or adult testis) (see Methods, Table 1 and Additional file 4). It should be noted that the 'embryonic general' class was generated from whole-body tissue (including the gonads), and also that in the generation of the embryonic gonad EST libraries individuals were not sexed, and thus the ESTs reflect undifferentiated gonads pooled from both sexes [45].

**Figure 2:**
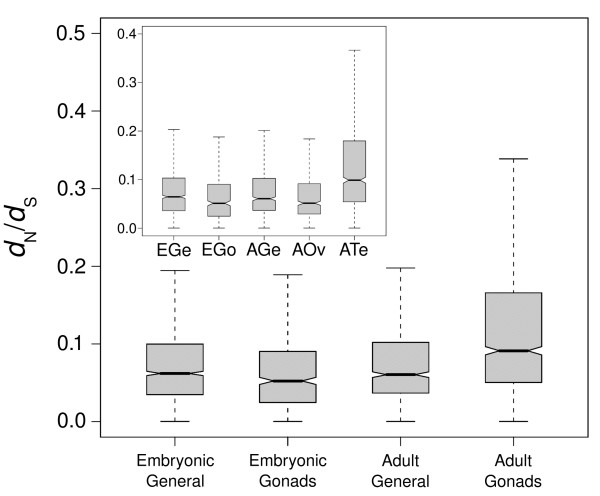
**Box plot of non-synonymous site divergence/synonymous site divergence (*d*_N_/*d*_S_) distributions for genes classified into gonadal or non-gonadal categories in the embryonic and adult stages based on expressed sequence tag (EST) data**. Classification of genes using a greater than twofold proportion of representation relative to other stages is shown. Inset indicates *d*_N_/*d*_S _distributions when adult stage gonads are separated into ovary and testis. Genes classified into the adult gonads category are evolving more rapidly than all other categories (*P *< 0.01) though this is only the case for the adult testis category when the gonads are classified separately. In contrast, genes classified in the embryonic gonads category are evolving less rapidly than all other categories (*P *< 0.05). Non-gonadal embryonic and adult tissues show no significant differences in their rates of evolution (*P *> 0.05). AGe = adult general; AOv = adult ovary; ATe = adult testis; EGe = embryonic general; EGo = embryonic gonads.

We found that genes classified as being expressed in combined adult gonads are evolving significantly more rapidly than all other stages in *d*_N_, *d*_S_, and *d*_N_/*d*_S _at all specificity thresholds (*P *< 0.01), with sole exception of the comparison between the *d*_S _of adult and embryonic gonads, which is non-significant (Figure 2, Additional files 10 and 11). When adult gonads are separated into either 'adult ovary' or 'adult testis', we found that only genes expressed in the testes showed an accelerated mean rate of evolution (*d*_N _and *d*_N_/*d*_S_) relative to other stages. Under those specificity thresholds where a significant difference in evolutionary rate was found between adult ovaries and other tissues, the mean rate of evolution of genes expressed in the ovaries was significantly lower than the other tissues (*P *< 0.05; Figure 2; Additional files 10 and 11). Contrary to what is observed in the adult, genes expressed in the embryonic gonads are evolving more slowly than non-gonadal tissues (*d*_N _and *d*_N_/*d*_S_; *P *< 0.05). We found no consistent significant differences in the rate of evolution between genes expressed in non-gonadal adult or embryonic tissue, supporting the results of previous studies indicating that gonadal expression plays a large role in explaining evolutionary rate differences in the adult stage [24,25].

As in the case of genes classified into specific stages, we performed pairwise comparisons of the proportion of genes showing evidence of positive selection for each tissue/stage and for each specificity threshold. Again, no comparisons were statistically significant after Bonferroni correction, with the sole exception that genes classified as unique to the adult testis have a significantly higher proportion of genes showing evidence of positive selection than genes classified as unique to the adult general category (χ^2 ^value = 8.76, df = 1, *P *= 0.0308) (Additional file 9).

### Gene interaction profiles during development

The development constraint hypothesis is predicated on the notion that development is coordinated by hierarchical genetic networks [15,16,46] and therefore features of early development are more likely to be constrained by selection against the deleterious pleiotropic effects of mutations [3,5,17]. A logical prediction of such theory is that genes involved in earlier stages of development should represent more central regulatory nodes and, on average, be involved in more interactions as a consequence. Using the BioGRID database [47] we obtained the total number of interactions associated with each gene from the EST-based dataset for which interaction information was available, resulting in a total of 4,422 genes involved in 34,462 interactions (Additional file 12). We found a significantly higher mean number of interactions per gene for genes specific to the embryonic stage as compared to the larval/pupal stage at no specificity threshold and a greater than twofold proportional representation threshold (Kruskal-Wallis permuted rank sum test, *P *= 0.0162 and 0.0003, respectively; Table 2; Additional files 12 and 13). The mean number of interactions was also higher for genes specific to the embryo as compared to the adult stage at most specificity thresholds (*P *< 0.05; Additional file 13). All other comparisons, including those between the larva/pupal and adult stages were non-significant. In order to minimize the potential effect of stage-specific ascertainment bias in the BioGRID database's genetic interaction data (that is, a greater proportion of genetic interaction experiments are likely performed during embryogenesis), we also performed the same analysis using only BioGRID's data on direct protein-protein interactions (yeast two-hybrid system; 4,092 genes involved in 23,712 interactions; Additional file 13). Our results remained qualitatively unchanged when using all yeast two-hybrid interaction data, however the majority of statistically significant comparisons disappeared when we limited our analysis only to the 'high-confidence interactions' as defined by Giot *et al*. [48] (2,736 genes involved in 5,589 interactions, Additional file 13), though the embryonic stage continues to show a higher mean number of interactions as compared to the larval/pupal stage at no specificity threshold and a greater than twofold proportional representation threshold (*P *= 0.0165 and 0.0471, respectively).

**Table 2:** Average number of interactions (95% confidence intervals (CIs)) per stage and per gonadal or non-gonadal categories in the embryonic and adult stages

	No threshold			Greater than twofold		
	
	n	Mean	95% CI	n	Mean	95% CI
Stage:						
Embryonic	2,159	8.495	7.884 to 9.107	1,415	8.913074205	8.097 to 9.729
Larval/pupal	858	7.401	6.566 to 8.240	439	6.760820046	5.611 to 7.915
Adult	1,405	6.955	6.291 to 7.619	926	6.457883369	5.713 to 7.201
Stage/tissue (adult gonads combined):						
Embryonic general	1,504	8.762	8.038 to 9.483	890	9.270	8.219 to 10.339
Embryonic gonad	609	8.612	7.176 to 10.039	331	7.254	6.039 to 8.476
Adult general	682	6.701	5.947 to 7.453	381	6.071	5.134 to 7.018
Adult gonad	973	6.864	6.128 to 7.603	635	6.543	5.639 to 7.453
Stage/tissue (adult gonads separated):						
Embryonic general	1,280	8.96171875	8.136 to 9.784	721	9.510402219	8.264 to 10.749
Embryonic gonad	520	8.726923077	7.086 to 10.346	263	7.566539924	6.060 to 9.077
Adult general	583	6.491467577	5.698 to 7.278	360	6.088888889	5.104 to 7.073
Adult ovary	713	7.553997195	6.683 to 8.419	334	7.508982036	6.298 to 8.722
Adult testis	763	6.804718218	6.024 to 7.590	539	6.769944341	5.758 to 7.769

Classifications using no threshold and greater than twofold proportional representation threshold are shown. (n) Indicates the number of genes in each category. Larval/pupal represents the pooled larval and pupal stages. The average number of interactions per stage was found to be statistically significantly higher in the embryonic stage as compare to the other two stages (*P *< 0.5). When the adult stage is separately classified into general, ovaries, and testis categories, only the general and testis categories show a statistically significantly fewer average number of interactions than the embryonic categories, when significant (Additional file 12).

When comparing the average number of interactions per gene between gonadal and non-gonadal tissues in the adult and embryonic stages, we observed significantly fewer interactions in both the adult non-gonad and adult gonad categories as compared to the embryonic general category at no specificity threshold and a greater than twofold proportion of representation threshold (*P *< 0.05). The embryonic gonad category showed a significantly higher mean number of interactions than both adult general and adult gonad categories only when no specificity threshold was used in classification (*P *< 0.05 after Bonferroni correction). No other pairwise comparisons of mean number of interactions per gene were statistically significantly different, including both within-stage comparisons of gonadal to non-gonadal tissue. When adult gonads were separated into either ovary or testis-specific genes, only genes classified as testis specific had significantly fewer mean interactions (*P *< 0.05 at no specificity threshold and a greater than twofold proportion of representation threshold). We then reanalyzed the data using only direct protein-protein interactions and again, results were qualitatively similar, though no pairwise comparison was statistically significant after Bonferroni correction when adult ovaries and testes were classified separately (with the sole exception of the embryo general category which shows a significantly higher mean number of interactions than the adult general category using no specificity threshold, *P *= 0.0180). Also similarly, limiting our analysis to 'high-confidence' interactions resulted in most of the significant comparisons to becoming non-significant, likely owing to the smaller total number of interactions as compared to the total dataset (Additional file 13).

Previous studies have demonstrated a significant negative correlation between the total number of interactions in which genes were involved and their rate of evolution [49,50]. Given our observation that increased stage specificity was positively correlated with evolutionary rate, we tested for a significant correlation between the number of stages in which a gene was represented and its number of interactions. We found a significant positive correlation between the number of stages in which genes are represented and the number of interactions in which they are involved (Kendall rank sum correlation test τ = 0.0848, *P *= 4.501 × 10^-12^).

## Discussion

Our study provides molecular confirmation of two different but non-mutually exclusive hypotheses seeking to explain Von Baer's 'Third Law', noting that morphological similarity among organisms tends to decrease over ontogeny [1]. Our findings consist of (1) evidence for stronger purifying selection during embryonic development as predicted by the modern developmental constraint hypothesis [3,5], (2) evidence for selection-driven accelerated divergence of genes in the adult stage, exemplified by those expressed in males, as predicted by Darwin [10], and (3) the existence of a temporal pleiotropy restricting the divergence of genes that are broadly expressed over the course of development.

### Expression patterns across the Drosophila phylogeny

All developmental and spatial representation of gene expression information in our study is based on data collected in *D. melanogaster*, therefore an underlying assumption is made that developmental and spatial expression patterns, or more specifically that the stage/tissue of highest expression level, do not vary significantly among species of the *D. melanogaster *subgroup. While several studies have shown considerable variation in expression levels between species at the adult stage [51,52], to our knowledge, there are no studies that have directly compared expression levels between species over development on a large scale. A study conducted by Rifkin *et al*. [53] found that approximately 17% of genes surveyed (2,193/12,866) had significant differences between species in the degree to which genes in expression pattern changed during the onset of metamorphosis in *D. melanogaster*, *D. simulans*, and *D. yakuba*. However, it is unclear if such changes imply that the stage of highest level of expression changes between species. Regardless, if patterns of expression varied considerably between the species used in our study, we would expect this to add noise to the evolutionary signals we observed rather than produce systematic biases in our dataset.

### Divergence patterns over development

The results of our analysis indicate that sequence follows the pattern observed in morphology over the course of development: we observed a positive gradient in the rates of divergence (*d*_N _and *d*_N_/*d*_S_) in subsequent stages of ontogeny (Figure 1, Additional file 1). However, in the case of the synonymous rate of substitution, *d*_S _is highest in adults and lowest in the larval/pupal stage (that is, larval/pupal < embryonic < adult) (Additional file 8). These observations are consistent with either (a) systematic variation in the level of codon usage bias between developmental stages, or (b) a systematic difference in the rate of mutation between stages of development. A recent study performed by Vicario *et al*. [38] confirmed that CUB varies significantly among developmental stages when estimated in both *D. melanogaster *and *D. pseudoobscura*. Furthermore, the pattern of variation in CUB that they observed (adult < embryonic < larval) mirrors the rate of synonymous substitution measured at each stage in our study, consistent with CUB being responsible for the patterns of variation in *d*_S _that we observe (that is, high CUB reduces *d*_S _by selecting against substitutions generating non-optimal codons) [54]. A similar analysis of the Codon Adaptation Index [55] using codonW [56] on our dataset agreed with Vicario *et al*.'s results (data not shown) [38]. While it is not possible to rule out the hypothesis of different mutation rates affecting genes expressed in different stages of ontogeny, the non-concordance between the patterns observed in the synonymous and non-synonymous rates of substitution, *d*_S _and *d*_N_, indicates that differential mutation rate alone is insufficient to explain the positive gradient of divergence in *d*_N _and *d*_N_/*d*_S _observed over ontogeny. However, a gradient in these divergence rates over development is predicted by both the developmental constraint and selection opportunity hypotheses and thus evidence supporting either or both will be considered below.

### Embryonic developmental constraint

Supporting the developmental constraint hypothesis, we observed an increased mean number of interactions per gene among genes showing their highest level of expression in the embryonic stage when compared to those specific to other stages (Table 2). This is consonant with the notion that the products of genes expressed in this stage are involved in a greater number of highly connected regulatory networks, and are thus constrained in their divergence due to the cascading effects of deleterious mutations [15,16]. We observed that genes classified as specific to the embryonic gonadal category were involved in significantly more interactions than those specific to the adult gonads, suggesting that lack of pleiotropy-mediated constraint may play some role in explaining the tolerance for evolutionary divergence of adult gonad specific genes when compared to those of other tissues and stages. This is particularly so in the case of the testis (Additional file 11).

A potential caveat to such analysis could occur if the majority of interaction studies in *Drosophila *were performed with the intention of identifying interactions in the embryo, thus biasing the data in favor of a greater number of embryo-specific gene interactors. However, when we limited our analysis to interactions derived from yeast two-hybrid experiments using gene predictions from the whole *Drosophila melanogaster *genome [48,57], our results remained qualitatively unchanged, suggesting that our dataset is not significantly biased towards any specific stage. It should be noted that the yeast two-hybrid technique is known to generate a large number of false positive predictions of protein-protein interactions (reviewed in [58]). However, in order for such false positives to have a significant effect in biasing our data, it would require that the whole genome yeast two-hybrid studies from which the interaction data are derived [48,57] preferentially produce false positives among genes expressed at their highest level in the embryonic stage. A large number of interactions in BioGRID's database are not derived from yeast two-hybrid studies, and limiting our analysis to these studies supports the results observed from the analysis of the entire dataset (data not shown). However, it is likely that interactions derived from these genetic studies are biased towards experiments conducted during embryogenesis, and thus such observations should be interpreted with caution.

Noting that very early ontogenic processes such as gastrulation can show considerable divergence among closely related species, Raff [5] suggested that developmental constraint may imperfectly reflect the sequence of organismal ontogeny, but rather that the constraining effects of pleiotropy should be highest during those developmental stages showing the least amount of modularity, or disassociation, between regulatory pathways. It is possible that, given the large scale morphogenesis that occurs during both embryogenesis and metamorphosis in *Drosophila*, more genes expressed during the embryonic and pupal stages occur in highly interconnected regulatory networks and thus are constrained by greater pleiotropy than those specific to the larval and adult stages. However, our analysis of the mean number of interactions of genes classified into the pooled larval and pupal stages found no significant difference when compared to genes classified into the adult stage (Table 2, Additional file 13). While this may be an effect of larval stage genes obscuring the signal of a greater number of interactions in the metamorphosis stage, this seems unlikely as under the strict predictions of the developmental constraint hypothesis, larval genes should be, on average, more conserved than those of the subsequent metamorphosis stage and therefore possibly involved in more interactions. Unfortunately, separate larval-derived and pupal-derived EST libraries will be required to answer such concerns. It should be noted that Arbeitman *et al*. [29] observed that the transcriptomes of the embryonic and pupal stages are more similar to one another than either is to the larval or adult, suggesting that many genes classified as embryonic specific may have important functions in metamorphosis.

### Selection opportunity and adult divergence

Unlike the developmental constraint hypothesis, which predicts that the gradient in divergence rates observed over ontogeny is produced by relaxed selective constraint occurring on genes expressed in later stages, Darwin's selection opportunity hypothesis argues that this gradient is driven by positive selection [10]. Unfortunately, an increase in *d*_N _and *d*_N_/*d*_S _over development, as we observed, is consistent with both positive selection and relaxed selective constraint. However, as part of the predictions of the selection opportunity hypothesis, we should also observe an increase in the proportion of positively selected genes in later stages of development [43]. When examining the proportion of genes showing evidence of positive selection among our three developmental stages, the differences between stages were not statistically significant (Additional file 9). It should be noted however, that the number of genes in our dataset showing significant evidence of positive selection was quite small (359 out of 7,180 genes classified under no specificity threshold) and may represent too limited a dataset from which to draw statistically meaningful conclusions. While this may suggest that our results do not support Darwin's hypothesis, it is interesting that our study of both EST and microarray-based datasets noted that the accelerated rate of evolution observed in the adult stage is explained by the rapid evolution of male-biased genes and, more specifically, those expressed in the testis (Figure 2, Additional files 1 and 2). This result is consistent with previous morphological studies conducted within the *D. melanogaster *species complex that found that sexual traits (for example, genital arch area, testes length and area) show consistent, statistically significant differences between species, whereas non-sexual traits (for example, wing length and width, tibia and femur length, and malpighian tubules length and area) do not [59]. Numerous studies have found that genes involved in sex and reproduction diverge rapidly under the effect of positive selection [60-64] and, more specifically, that genes with sex-biased expression show greater evidence of positive selection than non-sex biased genes [65,66]. Thus there appears to be evidence that the accelerated evolution observed in later stages of development is driven by unique selective pressures such as sexual selection (but see also [67,68] for examples of theory and empirical evidence suggesting relaxed selective constraint has a large effect in explaining the rapid evolution of genes with sex-limited expression).

Darwin's hypothesis that selection opportunity increases over the course of ontogeny also requires that the effects of selective pressure should only be observed at the stage in which the pressure occurs, and for which he presented secondary sexual traits as an example [10]. While few studies have analyzed the rate of evolution of embryonic genes [23-25], numerous analyses have shown that adult traits and genes involved in reproduction, particularly in male reproductive organs, often evolve at accelerated evolutionary rates when compared to most other tissues [12,36,41,60-65]. As expected, we observed that genes expressed in the pooled gonads of the adult fly are evolving more rapidly than non-gonadal adult tissue (Figure 2a, Additional file 11). In the case of the pooled embryonic gonads, under all specificity thresholds where the differences were statistically significant, genes classified as embryonic gonad specific are evolving less rapidly than whole embryonic tissue. Thus the situation of accelerated evolution of gonad specific genes in the adult is reversed in the embryo, suggesting that the selective forces occurring in the adult reproductive stage are acting primarily on genes expressed at that stage; or at least are not affecting the embryonic stage.

### Temporal pleiotropy and protein evolution

A negative correlation between breadth of gene expression and protein divergence has been observed in taxa as distant as primates and flies [40-42] suggesting the existence of a broadly applicable mechanism constraining the divergence of genes expressed in multiple tissues. The most plausible of such mechanisms is negative selection against the deleterious pleiotropic effects engendered from mutations occurring in highly connected genes [49,50,69]. Our data suggest that such a model should be extended to include temporal pleitotropy to the well supported spatial pleiotropy observed in previous studies. We observed a clear pattern of increasing evolutionary divergence (in both *d*_N _and *d*_N_/*d*_S_) with increasing stage specificity of representation (Figure 1, Additional file 7), suggesting that genes expressed ubiquitously over the course of development are subject to similar, pleiotropy-mediated evolutionary constraints as genes that are ubiquitously expressed across different tissue types [40-42]. Furthermore, our observation of a significant positive correlation between the number of stages at which genes were represented and the average number of interactions in which these genes are involved strongly suggests that temporally ubiquitously expressed genes are involved in a greater number of cellular and organismal functions than their stage specific counterparts, and could thus be under more restricted evolutionary divergence due to the large effect of deleterious mutations at these loci.

## Conclusion

**Figure 3:**
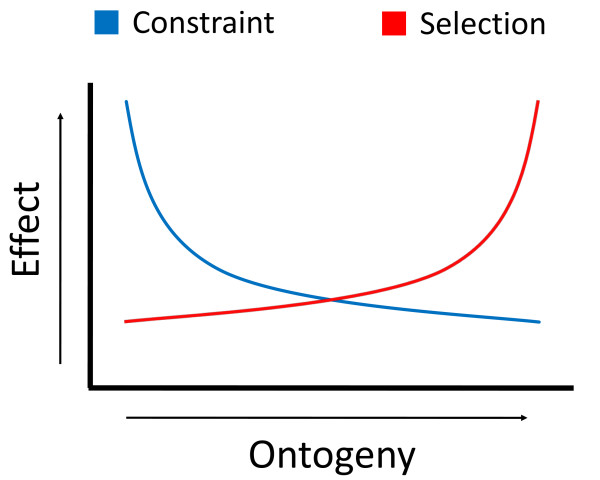
**Constraint-early/selection-late model of developmental divergence**. Reduced divergence rates of embryonic genes relative to those of later stages are explained by purifying selection against the deleterious pleiotropic effects of mutation. Later stage genes are not simply 'less constrained', but experience unique selective pressures, such as sexual selection, driving accelerated divergence.

In conclusion, we found support for both pleiotropy mediated developmental constraints, as well as Darwin's selection opportunity, having a significant impact on the rates of divergence of genes over the course of ontogeny in *Drosophila*. These hypotheses are not mutually exclusive, but rather may work in tandem, each primarily influencing different stages of development in order to explain the ontogenic patterns observed among species. Therefore, given our observations, we propose a 'constraint-early/opportunity-late' model of evolutionary divergence over ontogeny (Figure 3), such that the reduced divergence of early-expressed embryonic genes is primarily explained by strong purifying selection minimizing the deleterious pleiotropic consequences of mutation. The accelerated divergence of late-expressed adult genes is primarily explained by unique selective pressures driving their divergence at this stage. More data and the availability of separate larval and pupal stage specific representation information will be required in order to determine the relative contributions of constraint and selection in these mid-ontogenic stages. Finally, our data imply that we ignore a large amount of information about the evolutionary dynamics of gene divergence by studying spatial gene expression at only a single stage. Any holistic approach to understanding the evolutionary dynamics of gene divergence will have to take into account temporal pleiotropy in addition to spatial pleiotropy, and as such, more temporal information about gene expression will be required in order to generate a better understanding of evolutionary divergence in which both constraint and opportunity play a role.

## Methods

### Gene evolutionary rate estimates

All estimates of gene evolutionary rates were obtained from the *Drosophila *12 Genomes Consortium Sequencing/Annotation Project [33] according to their PAML estimates [44] performed on six species of the *D. melanogaster *group: *D. melanogaster*, *D. simulans*, *D. sechellia*, *D. yakuba*, *D. erecta*, and *D. ananassae *[42]. *d*_N_, *d*_S_, and *d*_N_/*d*_S _(ω in PAML) as calculated under model 0 were used in this analysis. For the EST library-based developmental profile (see below) the number of genes showing evidence of positive selection at each stage and for each stage/tissue category were obtained from the FDR corrected non-branch specific comparisons of models 7 and 8 in the *Drosophila *12 Genomes Consortium dataset [33].

### EST library-based developmental profile

We obtained information about the representation of all 7,180 genes in the *Drosophila *12 Genomes Consortium [33] dataset that were found in all stage-specific *D. melanogaster *EST libraries in the National Center for Biotechnology Information (NCBI) UniGene database (release version 53) [35,70]. EST libraries separately representing the larval and pupal stages were unavailable, therefore libraries were pooled into one of three developmental stage categories based on the stage from which they were generated: embryonic, larval/pupal, and adult. Genes were then classified into developmental stages based on the stage in which they showed their highest proportion of representation among sequenced ESTs (that is, the number of sequenced ESTs from each gene divided by the total number of ESTs sequenced in that stage's pooled libraries). Genes were reclassified into developmental stages using a series of arbitrarily chosen specificity thresholds, such that in order for a gene to be classified as specific to a stage, its highest proportion of representation had to occur at that stage and also exceed the proportion of representation at any other stage by a threshold of more than twofold, fourfold, or eightfold. Genes were also classified into a 'unique' category if they were represented only in libraries generated from a single stage, therefore producing a series of five separate sets of genes assigned to specific developmental stages (Table 1, Additional file 4).

EST libraries from the embryonic and adult stages were separated into those derived specifically from the gonads and those derived from whole embryos (including the gonads) in the case of the embryo, and from all other tissues (not including the gonads) in the case of the adult. Genes were then classified into embryonic general, embryonic gonads, adult general, and adult gonads as indicated above, using the same specificity thresholds. In the case of the adult stage, testis-derived and ovary-derived libraries were either classified together as 'adult gonads' or separated into 'adult testis' and 'adult ovary' categories. For the purposes of this comparison, all genes classified as larval/pupal-specific were ignored. The number of genes classified into each category and proportion of representation threshold from the EST analysis is shown in Table 1. In the comparison of adult and embryonic gonads and non-gonadal tissue, it is important to note that the numbers of genes classified into each category varies based on whether the adult gonads are combined or separated, especially at lower specificity thresholds, owing to the change in proportional representation introduced when the testis and ovary libraries are pooled.

### FatiGO validation of EST-based classification

We obtained NCBI 'CG' numbers for all stage classified genes for which they were available (7,027 genes) using the 'symbol: symbol synonyms' tag in Flybase's [71] batch download feature. In the case where a Flybase gene (FBgn) was associated with multiple CG numbers, the CG number presented under the 'annotation symbol' heading of that FBgn's 'gene report' page was used. The few duplicate CG numbers occurring due to multiple FBgns linking to the same CG number were not removed. These duplicates most likely result from the splitting of what was originally a single gene into two when genome projects are reannotated. The list of CG numbers classified as specific to each stage were compared to one another using FatiGO[36,37,72], searching for over-representation of GO-biological processes in *Drosophila melanogaster *using a two-tailed Fisher exact test without duplicate filtering. Only significantly over-represented terms at GO levels 3 and 4 were collected.

### Developmental profile of interactions

We collected protein and gene interaction data for the 4,422 genes from the EST dataset (Additional file 4) that were represented in the BioGRID database (release 2.0.36) [47,73]. The total number of interactions, irrespective of the experimental methodology used to obtain them, that each gene was involved in was compiled and used in the analysis. We also compiled a dataset limited only to those interactions derived from yeast two-hybrid experiments for the purpose of ascertaining potential artifacts generated by biased stage sampling of genetic interactions (see Results) (Additional file 12). Finally, we also analyzed the dataset using only 'high-confidence' yeast two-hybrid interactions as defined by Giot *et al*. [48] (that is, those interactions with a confidence score greater than 0.5).

### Statistics

All statistical analyses were performed using the R statistical package [74]. Permuted Kruskal-Wallis rank sum tests and 95% confidence intervals were computed using 10,000 permutations of the data using the 'coin' and 'boot' packages, respectively. Pairwise comparisons of the proportion of genes under positive selection were performed using χ^2 ^tests. A Bonferroni correction for the effect of multiple tests was applied to all pairwise comparisons.

## Abbreviations

CI: confidence interval; CUB: codon usage bias; *d*_N_: number of non-synonymous substitutions per non-synonymous site; *d*_S_: number of synonymous substitutions per synonymous site; EST: expressed sequence tag; MYD: million years diverged; NCBI: National Center for Biotechnology Information; NSERC: Natural Sciences and Engineering Research Council of Canada; PAML: phylogenetic analysis by maximum likelihood.

## Authors' contributions

CGA, WH, and RSS conceived of the study and drafted the manuscript. CGA and WH collected data for analysis. CGA carried out the data analysis and interpretation.

## Supplementary Material

Additional file 1 Reanalysis of Arbeitman *et al*. microarray dataset using the stage classification approach used on the expressed sequence tag (EST) dataset. Click here for file

Additional file 2 Stage classification of Arbeitman *et al*.'s expression data [29]. Click here for file

Additional file 3 Pairwise permuted Kruskal-Wallis rank sum tests of the significance of the difference between the distributions of non-synonymous site divergence (*d*_N_), synonymous site divergence (*d*_S_), and *d*_N_/*d*_S _for different stages based on arbitrary expression threshold cut-offs of Arbeitman *et al*.'s expression data [29]. Click here for file

Additional file 4 Stage classification of the expressed sequence tag (EST) dataset. Click here for file

Additional file 5 Gene ontology terms over-represented in pairwise comparisons between the three stage classifications at each classification threshold using FatiGO. Click here for file

Additional file 6 Pairwise permuted Kruskal-Wallis rank sum tests of the significance of the distribution between different stages for *d*_N_, *d*_S_, and *d*_N_/*d*_S _based on arbitrary cut-offs of expressed sequence tag (EST) library representation data. Click here for file

Additional file 7 Pairwise permuted Kruskal-Wallis rank sum tests of the significance of the distribution between different arbitrary specificity thresholds within each stage for non-synonymous site divergence (*d*_N_), synonymous site divergence (*d*_S_), and *d*_N_/*d*_S _based on expressed sequence tag (EST) library representation data. Click here for file

Additional file 8 Average non-synonymous site divergence (*d*_N_) and synonymous site divergence (*d*_S_) values for genes classified into developmental stages based on expressed sequence tag (EST) data. Click here for file

Additional file 9 χ^2 ^Tests of the significance of the difference in the proportion of genes showing significant evidence of positive selection in pairwise comparisons between expressed sequence tag (EST) data classified stages and stages/organs for each specificity threshold. Click here for file

Additional file 10 Box plots of non-synonymous site divergence (*d*_N_), synonymous site divergence (*d*_S_), and *d*_N_/*d*_S _distributions for genes classified into gonadal or non-gonadal categories in the embryonic and adult stages using five specificity thresholds based on expressed sequence tag (EST) data. Click here for file

Additional file 11 Pairwise permuted Kruskal-Wallis rank sum tests of the significance of the distribution between gonadal and non-gonadal tissue at adult and embryonic stages for non-synonymous site divergence (*d*_N_), synonymous site divergence (*d*_S_), and *d*_N_/*d*_S _based on arbitrary cut-offs of expressed sequence tag (EST) library representation data. Click here for file

Additional file 12 Genes from the expressed sequence tag (EST) dataset for which BioGRID interaction information was available. Click here for file

Additional file 13 Pairwise permuted Kruskal-Wallis rank sum tests of the significance of the difference in the distribution of the number of interactions per gene in comparisons between expressed sequence tag (EST) data classified stages and stages/organs for each specificity threshold. Click here for file
