# Supplementary material for: Ontogeny and phylogeny: molecular signatures of selection, constraint, and temporal pleiotropy in the development of Drosophila
Source: BMC Biol. 2009 Jul 21;7:42. doi: 10.1186/1741-7007-7-42 (PMC2722573; doi:10.1186/1741-7007-7-42)
Supplement: Additional file 1 — Reanalysis of Arbeitman et al. microarray dataset using the stage classification approach used on the expressed sequence tag (EST) dataset. [file 1741-7007-7-42-S1.pdf]

Supplementary File 1 – Reanalysis of Arbeitman et al. microarray dataset using the stage classification approach used on the EST dataset.

Davis et al. [25] used the results of a cDNA microarray based study of expression of 4,028 genes over the course of *D. melanogaster* development [29] and found that expression level in the late embryonic stage was negatively correlated with gene divergence between *D. melanogaster* and *D. pseudoobscura*, while it was positively correlated with gene divergence in later stages. We sought to test the generality of their results using divergence estimates obtained from orthologs within the more closely related *D. melanogaster* group [33] and using a similar approach as that described in our analysis of the EST dataset (see Methods).

Data collection

We obtained raw expression datasets from Arbeitman et al.'s study of gene expression over the course of *Drosophila* development [29] (Gene Expression Omnibus Accession #GDS191) and concatenated all of the non tudor mutant fly array values for each of the 2,168 genes on the array for which we could obtain FlyBase [71] identifiers (FBgns) represented among the *Drosophila* 12 Genomes Consortium data [33]. Each gene was classified into 1 of either 4 developmental stages (embryonic, larval, pupal, and adult) or 5 developmental stages (where adult is separated into either adult male or adult female) based on the stage at which it shows its highest level of expression (Supplementary Table 9). The entire dataset of genes was then re-classified into the same stages using arbitrarily chosen specificity thresholds such that in order for a gene to be classified as specific to a stage, its highest level of expression had to occur at that stage and exceed the next highest level of expression measured at any other stage by more than a 0.5 fold or 1.0 fold expression difference. The number of genes classified into each category and expression threshold is shown in Supplementary File 1 - Table 3. Note that the 'adult male' and 'adult female' categories do not add up to the value given in

the 'adult combined' category owing to genes whose expression level is similar in both males and females and thus cannot be classified as specific to either category at given specificity thresholds.

Reanalysis of Arbeitman et al.'s microarray data

In their analysis of Arbeitman et al.'s microarray based developmental profile of expression [29], Davis et al. found that expression level in the late embryonic stage was negatively correlated with gene divergence between *D. melanogaster* and *D. pseudoobscura*, while it was positively correlated with gene divergence in the adult male [25].

We found significant differences in terms of substitution rates (d_N, d_S, and d_N/d_S) between stages under all thresholds (Kruskal-Wallis permuted rank sum test, $p < 0.01$ in all cases; Supplementary File 1 - Table 4). To examine the evolutionary dynamics of specific stages, we performed Bonferroni corrected pairwise Kruskal-Wallis permuted rank sum test comparisons of the distributions of rates of sequence divergence between all stages for each of the three specificity thresholds (i.e., no threshold, greater than 0.5 fold, and greater than 1.0 fold expression difference) (Supplementary Table 10). Genes expressed at their highest level in adults evolve more rapidly than those expressed during embryogenesis at all three specificity thresholds in terms of d_N, and d_N/d_S ($p < 2.2 \times 10^{-16}$; Supplementary File 1 - Table 5; Supplementary Table 10). Adult genes also evolve more rapidly than those of the pupal stage at all three specificity thresholds in terms of d_N and d_N/d_S ($p < 0.05$; Supplementary File 1 - Table 5; Supplementary Table 10). However, we found no significant differences in the comparisons of the distributions of d_N or d_N/d_S between other stages, except for a higher d_N in the adult as compared to the larval stage at no specificity threshold ($p = 0.0377$).

When we separated genes classified into the adult stage into those expressed at higher levels in males or females, we found that only those classified as adult male specific were evolving more rapidly than earlier stages (Supplementary Table 10). In this case, the comparison between the adult male stage

and the larval stage was statistically significant in terms of the d_N and d_S at no expression threshold ($p < 2.2 \times 10^{-16}$ and $p = 0.0117$, respectively) and at a threshold of greater that a 0.5 fold expression difference ($p = 0.0234$ and 0.0364, respectively). The rate of evolution of genes classified as adult female specific was not statistically significantly different from that of earlier stages.

Comparison between analyses

Using correlation analysis Davis et al. [25] found a negative correlation between gene expression and sequence divergence (d_N) in the late embryo, while they observed a positive correlation in the adult male. This supports the notion that genes follow the same pattern observed in morphology over development: genes expressed primarily in earlier stages are more conserved than those expressed in later stages. Our reanalysis of the data support accelerated sequence divergence in genes expressed primarily in adult males relative to earlier developmental stages (as well as relative to genes expressed primarily in adult females) (Supplementary table 10). The exception to this observation is the comparison between the adult and larval stage, where adult divergence was only significantly greater in the comparison of the d_N using no specificity threshold ($p = 0.0377$). It should be noted however, that there were fewer genes classified as being specific to the larval stage than the other developmental stages (Supplementary File 1 - Table 3), and this is likely limiting the statistical power of the analysis, especially at greater specificity thresholds. Our observation of fewer genes having their highest level of expression in the larval stage is somewhat puzzling given recent findings that codon usage bias (CUB) is highest in the larval stage, relative to other stages, in both *D. melanogaster* and *D. pseudoobscura* [38]. We may expect that if CUB is being maintained due to selection for translational efficiency, the stage with the highest CUB would have an overabundance of transcripts relative to other stages. It is possible that the limited size of our dataset (2,186 genes in the *Drosophila* 12 Genomes Consortium dataset [33]) or the limited number of genes probed in the original microarray experiment (4,028 or ~32% of the *D. melanogaster* predicted *D. melanogaster* transcriptome [75]) is leading to a bias in the

number of genes that are expressed at their highest level in the larval and pupal stages.

In general, we also noted and absence of significant difference in the mean rates of divergence, in terms of d_N and d_N/d_S, of genes in comparisons among earlier stages (i.e., embryonic, larval, and pupal). Given that our analysis of the larger, EST based dataset revealed statistically significant differences in the rates of evolution between classified as being specific to the embryonic and larval/pupal stages, it is likely that the lack of significant differences among microarray based stage classifications reflects the small size of the dataset rather than the absence of actual differences. We did note a significantly lower d_S in the pupal stage as compared to the embryonic stage, which supports Vicario et al.'s observation that CUB is higher in the pupal stage than in the embryonic stage [38]. Therefore the lower d_S in the embryo likely reflects reduced divergence due to selection for optimal codons [54].

Supplementary File 1 - Table 3. Number of genes classified into each stage and sex at the three expression specificity thresholds used to classify Arbeitman et al.'s [29] microarray data.

Stage	No Threshold	> 0.5 fold	> 1.0 fold
Embryonic	1262	848	433
Larval	118	38	8
Pupal	331	144	63
Adult Combined	444	258	163
Adult Male	339	199	118
Adult Female	104	21	12

Supplementary File 1 - Table 4: Permuted Kruskal-Wallis rank sum test results testing for significant differences between stages for d_N, d_S, and d_N/d_S based on Arbeitman et al.'s [29] data.

Specificity Threshold	Substitution Rate	*p* value	χ^2 value
None	d_N	< 2.2e-16	31.6924
	d_S	< 2.2e-16	43.4711
	d_N/d_S	0.0001	22.8441
0.5 fold	d_N	< 2.2e-16	60.7738
	d_S	< 2.2e-16	61.3126
	d_N/d_S	< 2.2e-16	32.4076
1.0 fold	d_N	< 2.2e-16	63.7833
	d_S	< 2.2e-16	51.8955
	d_N/d_S	< 2.2e-16	39.9070

Supplementary File 1 - Table 5. Average substitution rates (95% CI limits) per stage at no, greater than 0.5 fold, and greater than 1.0 fold expression difference thresholds based on Arbeitman et al.'s [29] expression data. The adult stage is presented when classified as a pool of both sexes as well as when both sexes are classified separately.

Stage		No threshold		Greater than 0.5 fold		Greater than 1.0 fold	
		mean	95% CI	mean	95% CI	mean	95% CI
Embryonic	d_N	0.116	(0.111 – 0.122)	0.121	(0.114 – 0.129)	0.124	(0.112 – 0.133)
	d_S	1.800	(1.769 – 1.832)	1.815	(1.777 – 1.854)	1.835	(1.780 – 1.886)
	d_N/d_S	0.067	(0.064 – 0.071)	0.070	(0.065 – 0.074)	0.070	(0.063 – 0.076)
Larval	d_N	0.1169	(0.098 – 0.136)	0.129	(0.088 – 0.169)	0.225	(0.104 – 0.347)
	d_S	1.7220	(1.627 – 1.826)	1.722	(1.545 – 1.900)	1.954	(1.515 – 2.389)
	d_N/d_S	0.0718	(0.058 – 0.086)	0.074	(0.053 – 0.095)	0.107	(0.067 – 0.146)
Pupal	d_N	0.1152	(0.104 – 0.127)	0.113	(0.094 – 0.131)	0.127	(0.097 – 0.162)
	d_S	1.6310	(1.573 – 1.689)	1.609	(1.523 – 1.696)	1.538	(1.412 – 1.665)
	d_N/d_S	0.0728	(0.065 – 0.081)	0.072	(0.060 – 0.085)	0.085	(0.060 – 0.109)
Adult (combined)	d_N	0.1626	(0.149 – 0.177)	0.193	(0.174 – 0.214)	0.214	(0.188 – 0.241)
	d_S	2.0010	(1.757 – 2.248)	2.209	(1.783 – 2.631)	2.389	(1.708 – 3.056)
	d_N/d_S	0.0865	(0.079 – 0.094)	0.098	(0.087 – 0.109)	0.107	(0.093 – 0.121)
Adult female	d_N	0.1074	(0.089 – 0.126)	0.121	(0.063 – 0.179)	0.098	(0.055 – 0.142)
	d_S	1.6840	(1.586 – 1.783)	1.812	(1.589 – 2.038)	1.897	(1.577 – 2.218)
	d_N/d_S	0.0646	(0.053 – 0.076)	0.067	(0.034 – 0.100)	0.054	(0.030 – 0.077)
Adult male	d_N	0.1798	(0.163 – 0.197)	0.203	(0.180 – 0.226)	0.227	(0.196 – 0.259)
	d_S	2.1000	(1.782 – 2.420)	2.322	(1.782 – 2.861)	2.543	(1.638 – 3.451)
	d_N/d_S	0.0934	(0.084 – 0.103)	0.102	(0.090 – 0.114)	0.113	(0.096 – 0.130)
