# Supplementary material for: Ontogeny and phylogeny: molecular signatures of selection, constraint, and temporal pleiotropy in the development of Drosophila
Source: BMC Biol. 2009 Jul 21;7:42. doi: 10.1186/1741-7007-7-42 (PMC2722573; doi:10.1186/1741-7007-7-42)
Supplement: Additional file 8 — Average non-synonymous site divergence (dN) and synonymous site divergence (dS) values for genes classified into developmental stages based on expressed sequence tag (EST) data. [file 1741-7007-7-42-S8.pdf]

Supplementary File 2 - Average A) d_N and B) d_S values for genes classified into developmental stages based on EST data. Averages are shown with permuted 95% confidence intervals for each specificity threshold: (from left to right, in increasing contrast) No specificity threshold, greater than two-fold representation, greater than four-fold representation, greater than eight-fold representation, and unique to that stage.

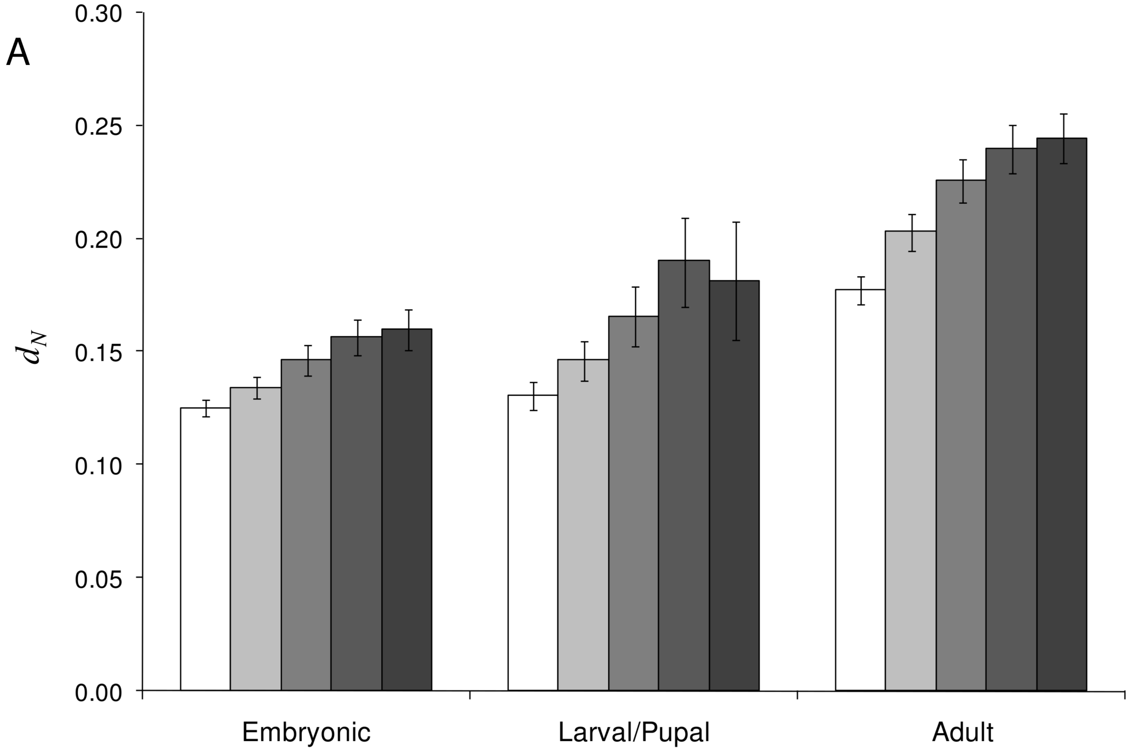

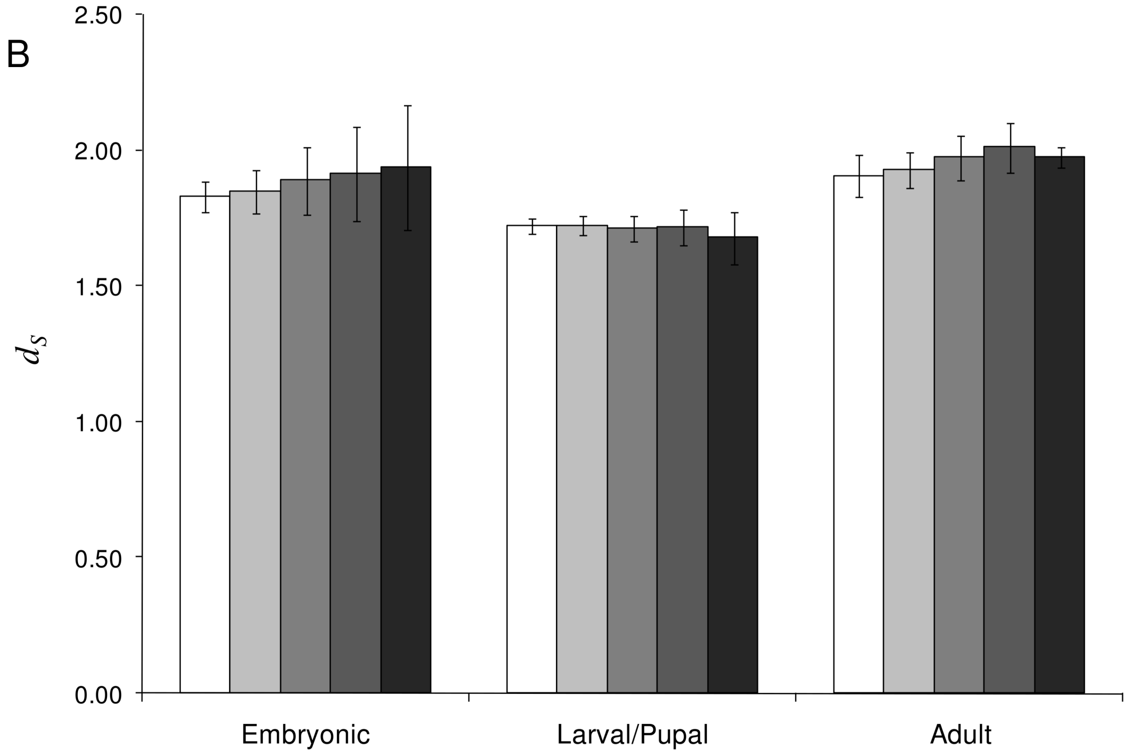
B
2.50
2.00
1.50
1.00
0.50
0.00
d_S
Embryonic
Larval/Pupal
Adult
