# Supplementary material for: Ontogeny and phylogeny: molecular signatures of selection, constraint, and temporal pleiotropy in the development of Drosophila
Source: BMC Biol. 2009 Jul 21;7:42. doi: 10.1186/1741-7007-7-42 (PMC2722573; doi:10.1186/1741-7007-7-42)
Supplement: Additional file 10 — Box plots of non-synonymous site divergence (dN), synonymous site divergence (dS), and dN/dS distributions for genes classified into gonadal or non-gonadal categories in the embryonic and adult stages using five specificity thresholds based on expressed sequence tag (EST) data. [file 1741-7007-7-42-S10.pdf]

Supplementary File 3 - Box plots of d_N, d_S, and d_N/d_S distributions for genes classified into gonadal or non-gonadal categories in the embryonic and adult stages based on EST data. Distributions are shown for each specificity threshold: (from left to right, in increasing contrast) No specificity threshold, greater than 2-fold, 4-fold, or 8-fold proportion of representation relative to other stages/tissues, and unique to a single developmental stage/tissue. Box plots were produced both by combining the adult gonads (A, B, C) as well as by separating ovary and testis into distinct categories (D, E, F) (see Methods).

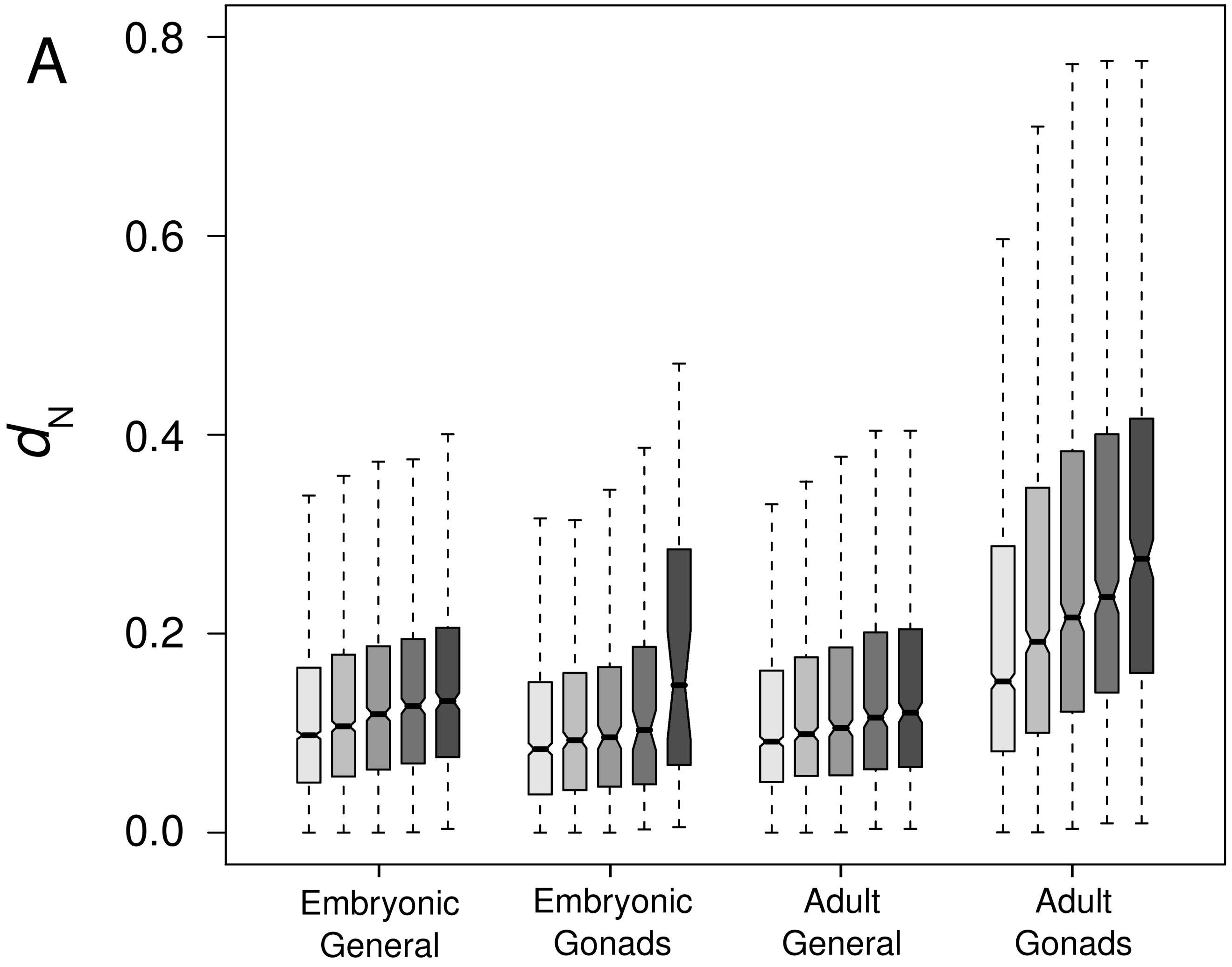

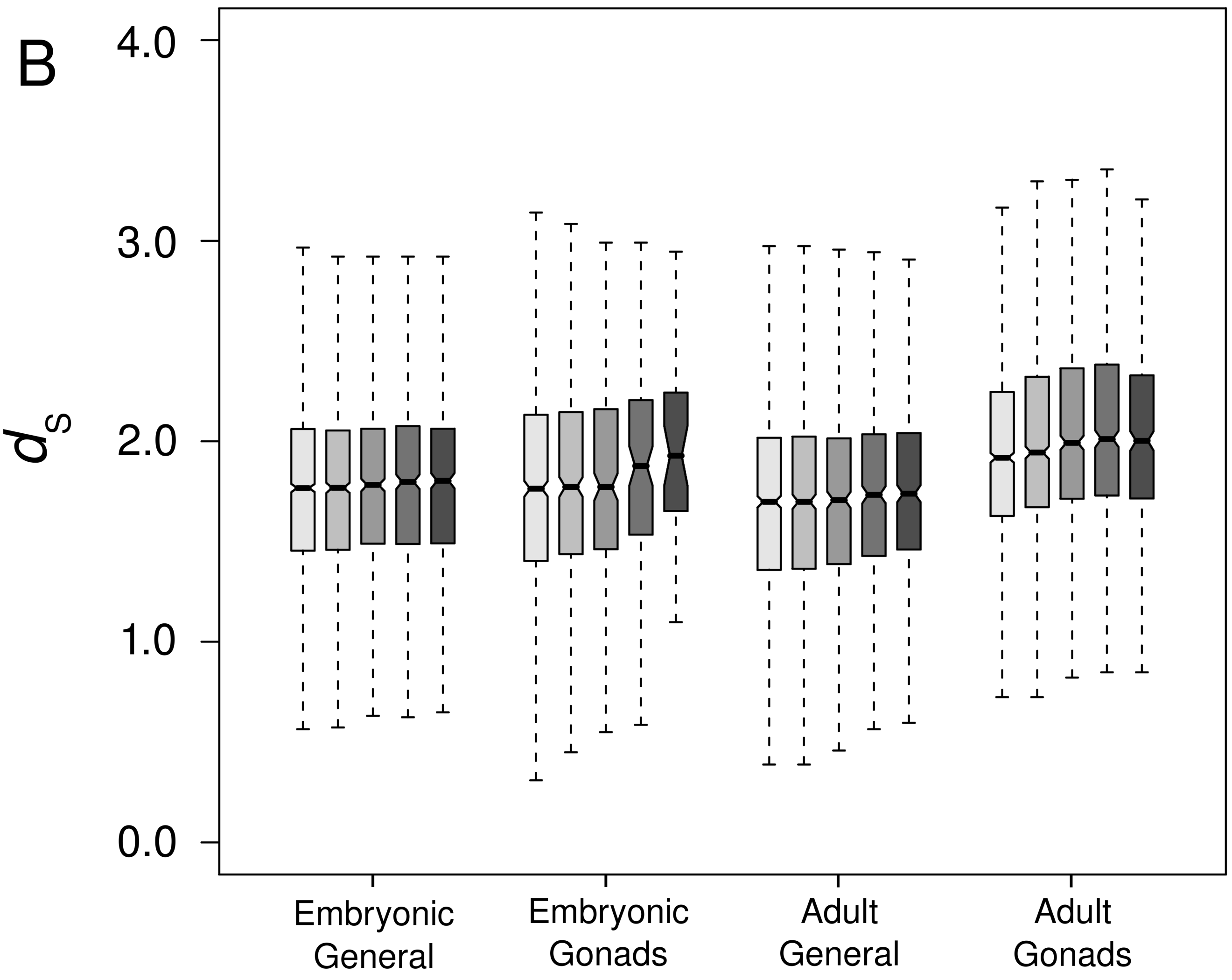
B
4.0
3.0
2.0
1.0
0.0
d_S
Embryonic General
Embryonic Gonads
Adult General
Adult Gonads

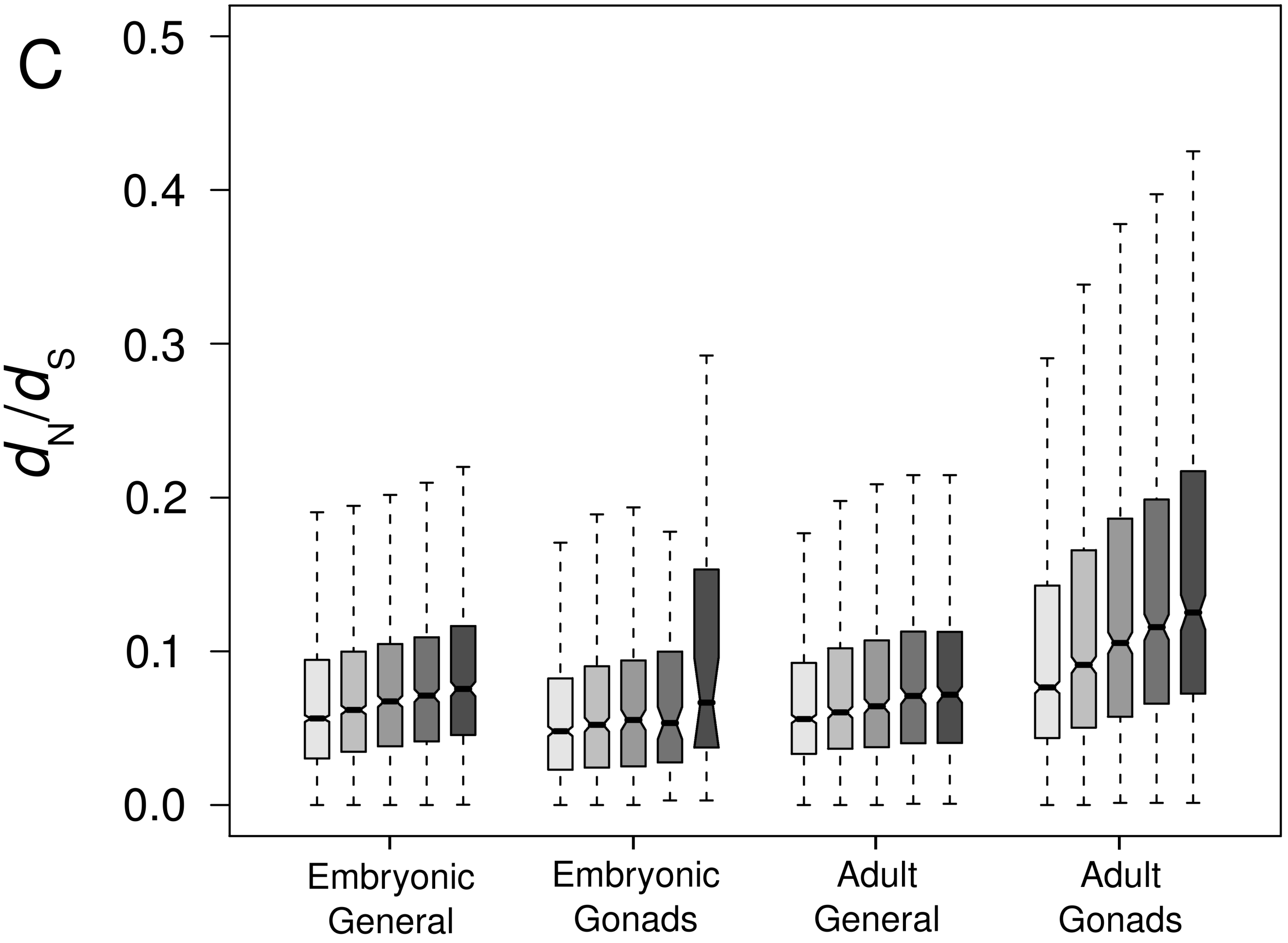

C
0.5
0.4
0.3
0.2
0.1
0.0
d_N/d_S
Embryonic General
Embryonic Gonads
Adult General
Adult Gonads

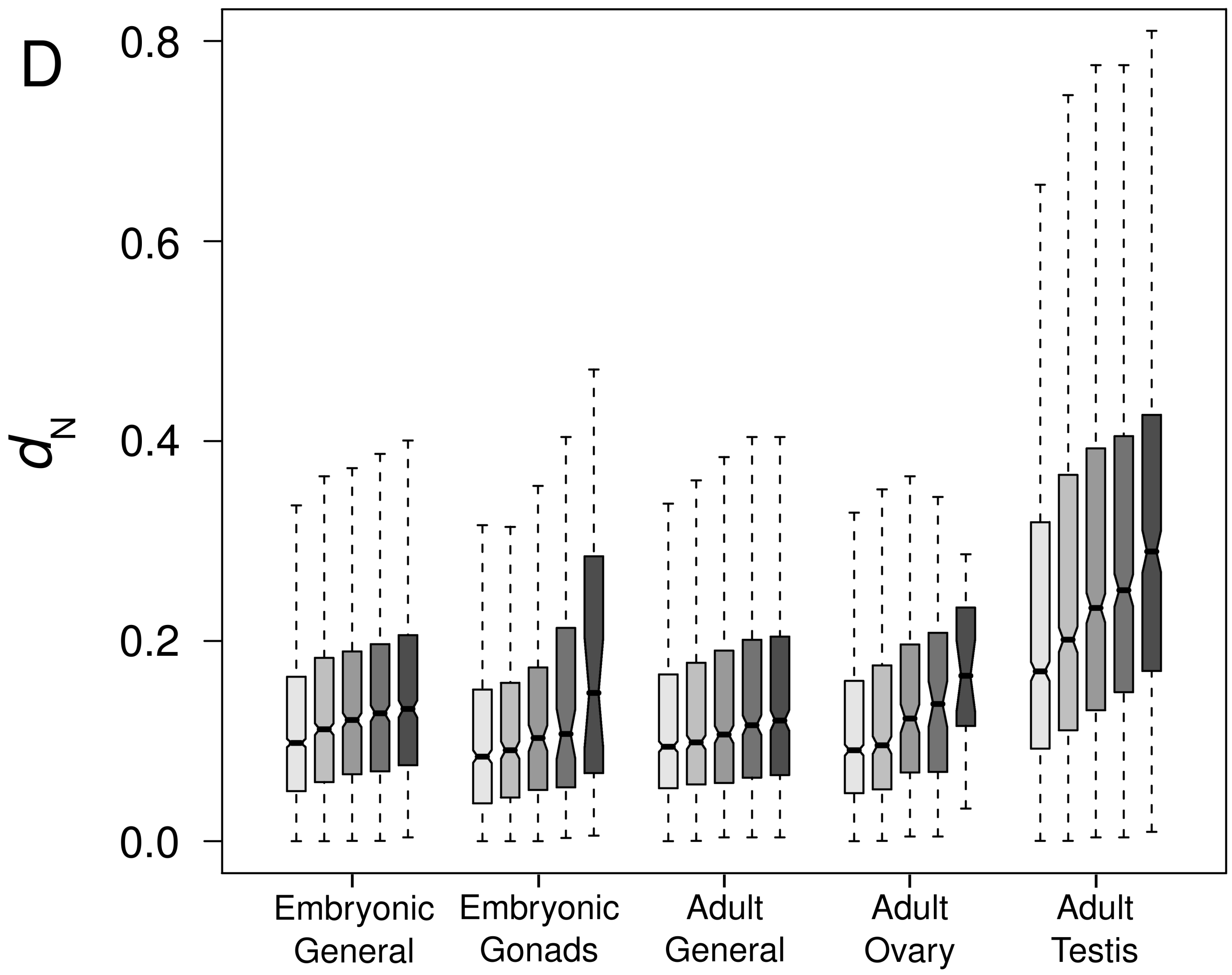
D
0.8
0.6
0.4
0.2
0.0
d_N
Embryonic General
Embryonic Gonads
Adult General
Adult Ovary
Adult Testis

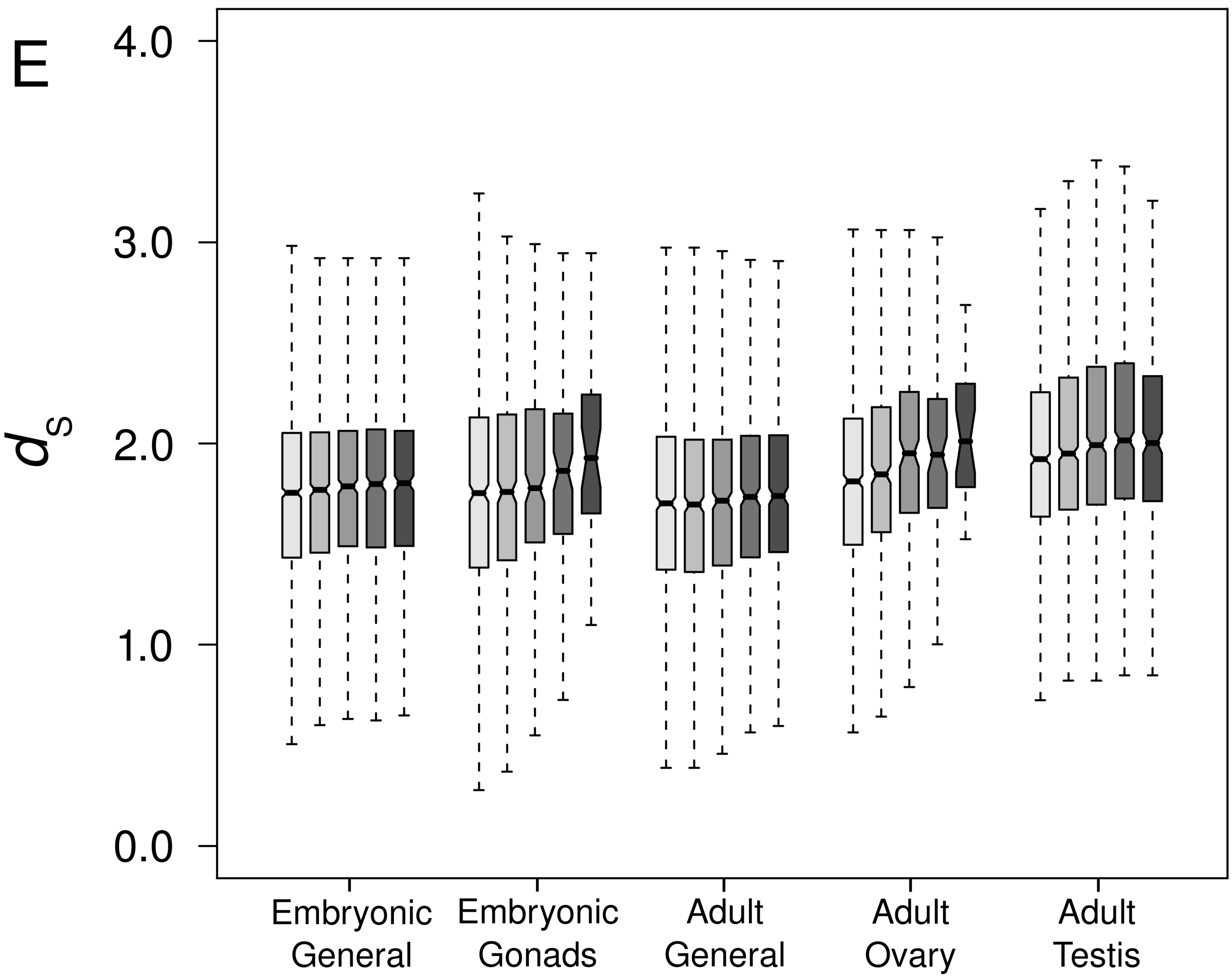

E
4.0
3.0
2.0
1.0
0.0
d_S
Embryonic General
Embryonic Gonads
Adult General
Adult Ovary
Adult Testis

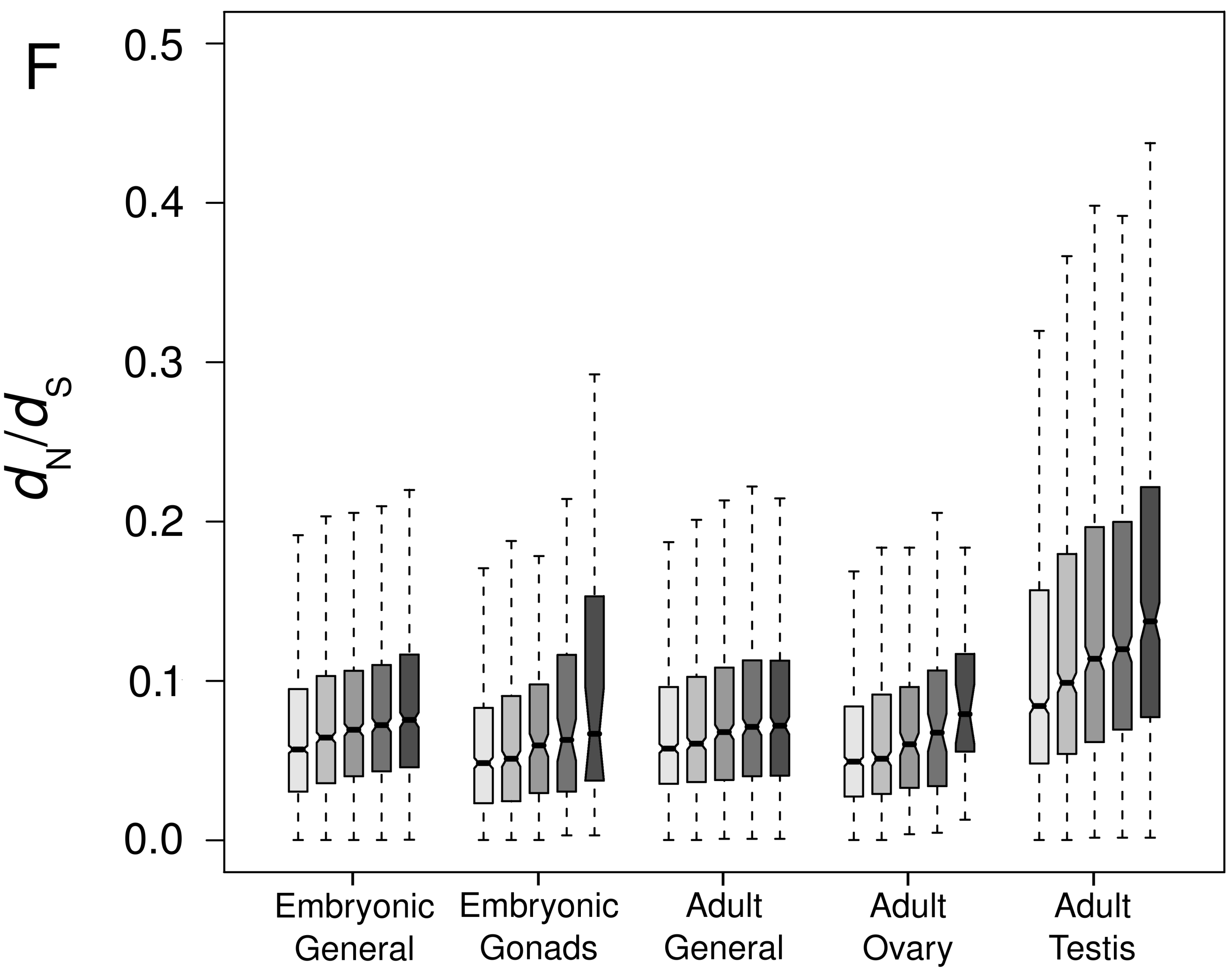
F
0.5
0.4
0.3
0.2
0.1
0.0
d_N/d_S
Embryonic General
Embryonic Gonads
Adult General
Adult Ovary
Adult Testis
